# Community pharmacists’ preparedness in irritable bowel syndrome management: A descriptive, community-based study on awareness, perceptions, and clinical support practices in Qassim, Saudi Arabia

**DOI:** 10.1097/MD.0000000000049312

**Published:** 2026-07-03

**Authors:** Mohammed Saif Anaam, Saeed Obaid Alfadly, Abdulmajeed Alqasoumi, Mohammed Almunef, Sulaiman Ibrahim Alsohaim, Suhaj Abdulsalim, Khalid Siddeeg, Bader Abdulah Al Harbi, Bader Theyab AL Mutairi, Waleed M. Altowayan

**Affiliations:** a Department of Pharmacy Practice, College of Pharmacy, Qassim University, Buraydah, Saudi Arabia; b Pharmacy Department, College of Medicine and Health Sciences, Al-Arab University, Mukalla, Yemen; c Department of Pharmacy, College of Medicine and Health Science, Hadhramout University, Mukalla, Yemen; d College of Pharmacy, Qassim University, Buraydah, Saudi Arabia.

**Keywords:** community pharmacists, cross-sectional study, irritable bowel syndrome, preparedness, quality of life

## Abstract

Irritable bowel syndrome (IBS) is a prevalent chronic gastrointestinal disorder affecting nearly 20% of the global population, with symptoms including constipation, diarrhea, bloating, and abdominal pain. As frontline healthcare providers, community pharmacists play a pivotal role in IBS management through medication guidance and patient education. However, their understanding, perceptions, and practices regarding IBS remain understudied in many regions, including Saudi Arabia. This study aimed to evaluate community pharmacists’ preparedness for IBS management by assessing their awareness, perceptions, and clinical support practices in the Qassim region of Saudi Arabia. A descriptive, cross-sectional study was conducted among community pharmacists in Qassim (May–August 2024) using a self-administered, 35-item questionnaire. The validated instrument assessed demographics, IBS awareness, perceptions, and clinical practices via closed-ended questions. Descriptive statistics were expressed as frequencies and percentages. Inferential analysis was performed using the Mann–Whitney *U* test for 2-group comparisons and the Kruskal–Wallis *H* test for comparisons involving 3 or more groups, with statistical significance set at *P* < .05. Of the 67 participants, 62.7% were male. The median age among all participants was 34 years (interquartile range = 32–38). While 61.2% of the participants demonstrated adequate IBS awareness, 38.8% had significant gaps. Notably, 77.6% recognized IBS as a quality-of-life-impairing condition, whereas 22.4% underestimated its clinical significance. Perceptions were largely constructive (64.2%), although 35.8% held negative views. Strikingly, 74.6% correctly identified IBS as more common than colorectal cancer, highlighting awareness of its prevalence. Awareness was significantly associated with higher education level (*P* = .041) but not with years of experience, sex, or nationality. In total, 52.65% of the pharmacists demonstrated adequate clinical preparedness for IBS management. While the majority of Qassim community pharmacists possess foundational competence for IBS counseling, the study reveals critical gaps in recognizing warning signs and applying evidence-based pharmacotherapy. These findings underscore the necessity of targeted educational interventions and continuing professional development programs to enhance pharmacists’ preparedness.

## 1. Introduction

Irritable bowel syndrome (IBS) is a chronic functional gastrointestinal disorder characterized by abdominal pain and altered bowel habits. It is a highly prevalent condition, with an estimated global prevalence ranging from 5% to 10%, as supported by recent studies.^[1,2]^ Research from various regions of Saudi Arabia indicates that the local prevalence varies considerably, ranging from 8% to 40%.^[3]^ Furthermore, this condition is associated with a significant economic burden stemming from both direct and indirect healthcare costs.^[4]^

In the United States alone, IBS accounted for over 2 million ambulatory care visits in 2010.^[5]^ More recently, a comprehensive meta-analysis by Arnaout et al^[6]^ estimated that direct medical costs associated with IBS in the United States range from $1.5 to $10 billion annually, excluding prescriptions and over-the-counter medications, with the condition accounting for approximately 15% of primary care referrals to gastroenterologists worldwide.^[6]^

This condition represents a significant clinical challenge, with 40% to 60% of patients experiencing comorbid psychological disturbances, such as anxiety and depression, further complicating disease management.^[7]^ Patients typically present with cramping abdominal pain, most commonly localized to the lower left quadrant, along with fluctuating symptoms that often lead to repeated healthcare consultations. The absence of definitive diagnostic markers and the variable nature of symptoms frequently result in initial self-medication with over-the-counter products before proper diagnosis.^[8]^

The elevated burden of IBS is not unique to Saudi Arabia but appears to be a regional phenomenon across the Middle East and other high-income developing countries. A large multicenter cross-sectional study conducted by Arnaout et al^[6]^ across 15 low- and middle-income countries revealed that IBS prevalence is substantially higher in these nations (mean = 25.2%, range 6.2%–44.2%) than in high-income countries, with particularly high rates observed in the Middle East.^[6]^ Within the Arab world specifically, a recent systematic review by Almansour^[9]^ encompassing 52 studies from 7 countries identified the highest prevalence rates in Saudi Arabia, Lebanon, and Jordan.^[9]^ Key risk factors identified across these populations – including family history, anxiety, depression, dietary changes, and sedentary lifestyles – mirror those observed in the Saudi population, suggesting shared etiological pathways. Indeed, depression, anxiety, family history, female sex, and specific dietary patterns (higher energy and carbohydrate intake) have been identified as significant risk factors specifically in the Saudi population.^[10]^

The pathophysiology of IBS remains incompletely understood, although recent advances have identified potential mechanisms involving gut-brain axis dysregulation, visceral hypersensitivity, and altered gut motility.^[11]^ Although IBS does not increase mortality risk, its substantial socioeconomic burden stems from increased healthcare utilization, reduced work productivity, and impaired quality of life.^[12]^

Current therapeutic approaches primarily focus on symptom management rather than cure, with limited patient satisfaction.^[8]^ This treatment gap underscores the critical role of healthcare providers, particularly community pharmacists, who serve as accessible frontline healthcare professionals. Numerous studies have demonstrated the effectiveness of pharmacists in improving medication adherence, providing patient education, and optimizing therapeutic outcomes for patients with gastrointestinal disorders, including IBS, as well as other chronic conditions such as diabetes and hypertension.^[13,14]^ Their unique position in the healthcare system enables ongoing monitoring and support for chronic conditions, such as IBS. Despite this potential, limited data exist regarding community pharmacists’ preparedness for IBS management in Saudi Arabia. Therefore, the present study aimed to evaluate community pharmacists’ preparedness for IBS management by assessing their awareness, perceptions, and clinical support practices in the Qassim region of Saudi Arabia, with the ultimate goal of enhancing patient care and identifying potential areas for educational intervention.

## 2. Materials and methods

### 2.1. Study period and place

From May to August 2024, a cross-sectional study was conducted among community pharmacists across 4 major cities in the Qassim region, Saudi Arabia: Buraidah, Unaizah, Ar Rass, and Al Mithnab. These cities were selected to provide a representative overview of the region. Buraidah, the regional capital, is the largest city, with the highest population density and concentration of healthcare facilities, including community pharmacies. Unaizah is the second-largest city in the region, with a well-developed healthcare infrastructure. Ar Rass is a significant city in the southern part of the region, with a moderate population and a corresponding number of community pharmacies serving as primary healthcare access points. Al Mithnab is a smaller city where community pharmacies play an even more central role in providing accessible healthcare advice. Including this range of cities – from the major urban center of Buraidah to the smaller community of Al Mithnab – allowed us to capture a broader spectrum of pharmacy practice settings and community pharmacist experiences across the Qassim region.

### 2.2. Study design

This descriptive, cross-sectional study was conducted using a validated, self-administered paper questionnaire to evaluate community pharmacists’ knowledge, perceptions, and clinical preparedness regarding the management of IBS.

### 2.3. Study population

Eligible participants included actively practicing community pharmacists in Qassim.

### 2.4. Inclusion/exclusion criteria

Participants were recruited via convenience sampling. The inclusion criteria were as follows: current employment as a community pharmacist and voluntary consent. Pharmacists who did not work in community settings or declined to participate were excluded.

### 2.5. Determination of sample size

The minimum required sample size was calculated using the Raosoft sample size calculator with the following parameters: 95% confidence level, 5% margin of error, and an anticipated response rate of 96%, based on a comparable KAP study among community pharmacists in the Qassim region.^[15]^ This calculation yielded an initial sample size estimate of 59 participants. To account for potentially incomplete or nonresponses, we increased this by 15%, resulting in a final target sample size of 67 community pharmacists. During the study period, 67 questionnaires were obtained, resulting in a response rate of 100%. Convenience sampling of community pharmacies was used, and each governorate – Buraidah, Unaizah, Ar Rass, and Al Mithnab – was divided into several areas, and the community pharmacies in these areas were visited. In cases where a pharmacy declined to participate or was unavailable, the next-nearest pharmacy was approached to ensure that the sample size was met.

### 2.6. Data collection questionnaire

Community pharmacists received a hard-copy, self-administered questionnaire during in-person visits across 4 Qassim cities (Buraidah, Unaizah, Ar Rass, and Al Mithnab). The researchers explained the study objectives before distribution. The 35-item survey aimed to evaluate community pharmacists’ preparedness for IBS management by assessing their awareness, perceptions, and clinical support practices. The questionnaire was developed by the research team based on a comprehensive review of current clinical guidelines for IBS management (e.g., the National Institute for Health and Care Excellence [UK] and the American College of Gastroenterology) and relevant literature on pharmacists’ roles in gastrointestinal health. To ensure its validity and reliability, the questionnaire was reviewed for content validity by a panel of 3 experts in clinical pharmacy and gastroenterology. The questionnaire combined closed-ended and multiple-choice questions with additional demographic items (sex, education level, and years of experience). A complete list of all awareness, perception, and practice items is provided in Tables 2 to 4, respectively. A 10-pharmacist pilot study confirmed question clarity without requiring modifications and confirmed face validity. Non-normally distributed domain scores were analyzed using median-based categorization: scores ≥ median indicated adequate awareness/constructive perceptions, whereas scores < median indicated inadequate awareness/limited perceptions.

### 2.7. Data analysis

Data analysis was performed using Statistical Package for the Social Sciences (SPSS, version 25; IBM Corp.).

Demographic and professional characteristics (sex, nationality, education level, and years of experience) were treated as categorical variables, as categorized in Table 1. Age was presented as the median and interquartile range because of its non-normal distribution. For inferential analysis, education level was collapsed into “Bachelor’s degree or less” (diploma/bachelor, n = 66) and “Postgraduate degree” (master, n = 1) to ensure adequate group sizes. Years of experience were analyzed in 3 groups: 1 to 5 years (early career), 6 to 10 years (mid-career), and >10 years (experienced). For the domain scores (awareness, perceptions, and clinical preparedness), the sum of correct or favorable responses was calculated. As these total scores were not normally distributed (assessed by the Kolmogorov–Smirnov test), participants were dichotomized based on the median score for each domain. Scores ≥ median were classified as adequate (awareness) or positive (perceptions/preparedness); scores < median were classified as inadequate or limited. Descriptive statistics, presented as frequencies and percentages, were used to summarize participant characteristics. Associations between variables were assessed using the Mann–Whitney *U* and the Kruskal–Wallis *H* tests. Statistical significance was set at *P* < .05.

**Table 1:** Demographic data of community pharmacists (n = 67).

Characteristics	Categories	N (%)
Sex	Male	42 (62.7)
	Female	25 (37.3)
Age	Median = 34 (IQR = 32–38)	
Nationality	Saudi	45 (67.2)
	Non-Saudi	22 (32.8)
Years of experience in pharmacy practice	1–5	46 (68.7)
	6–10	15 (22.4)
	>10	6 (9.0)
Level of education	Diploma	3 (4.5)
	Bachelor	63 (94.0)
	Master	1 (1.5)

IQR = interquartile range.

## 3. Results

### 3.1. Demographic characteristics

A total of 67 community pharmacists completed the questionnaire. The majority were male (62.7%, n = 42) and of Saudi nationality (67.2%, n = 45). The median age among all participants was 34 years (interquartile range = 32–38). Most participants held bachelor’s degrees (94%, n = 63); other demographic details are presented in Table 1.

### 3.2. Awareness of community pharmacists about IBS

Among the 67 participants, 77.6% (n = 52) correctly recognized IBS as a condition that significantly impaired quality of life, while 22.4% (n = 15) underestimated its clinical importance. Notably, 35.8% (n = 24) held the incorrect belief that IBS could reduce life expectancy, compared with 64.2% (n = 43) who disagreed. The majority (86.6%, n = 58) appropriately identified dietary factors as strongly associated with IBS, although only 22.4% (n = 15) correctly acknowledged the potential benefits of complementary therapies. Recognition rates for key clinical features were variable: 49.3% (n = 33) identified mucus in stool as a relevant symptom, whereas only 17.9% (n = 12) recognized anemia as an alarming sign. The results of the complete awareness assessment are presented in Table 2.

**Table 2:** Awareness of community pharmacists about irritable bowel syndrome (N = 67).

Questions	Responses (answers)	N (%)
IBS is a major condition that can have significant impairments to quality of life	True	52 (77.6)*
	False	15 (22.4)
In fact, IBS might decrease the life span of its victims	True	24 (35.8)
	False	43 (64.2)*
Compared with IBS, colon cancer is more common	True	24 (35.8)
	False	43 (64.2)*
Dietary factors, including food allergy and intolerance, are strongly associated with IBS	True	58 (86.6)*
	False	9 (13.4)
The main risk factors for IBS include genetic predisposition and prior bacterial or viral infection	True	62 (92.5)*
	False	5 (7.5)
IBS is often associated with psychological and emotional conditions	True	65 (97.0)*
	False	2 (3.0)
Other common symptoms of IBS include bloating, constipation, diarrhea, and abdominal pain	True	47 (89.1)*
	False	20 (10.9)
Modifying one’s diet may alleviate symptoms of IBS	True	63 (94.0)*
	False	4 (6.0)
Prescription drugs may help reduce the symptoms of IBS	True	65 (97.0)*
	False	2 (3.0)
Complementary therapies may make IBS symptoms better	True	52 (77.6)*
	False	15 (22.4)
With which of the following is IBS associated?	Blood in the stool	8 (11.9)
	Mucus in the stool	33 (49.3)*
	Dark-colored blood in the feces	11 (16.4)
	Diarrhea with oiliness	15 (22.4)
What are the more disturbing signs indicating that a person has IBS?	Reduction of weight	4 (6.0)
	Anemia	12 (17.9)*
	Female	37 (55.2)
	History of colorectal cancer in the family	14 (20.9)
Which one of the following drugs are frequently prescribed for IBS?	Famotidine	8 (11.9)
	Psyllium	29 (43.3)*
	Sulfasalazine	24 (35.8)
	NSAIDs	6 (9.0)

IBS = irritable bowel syndrome, NSAIDs **=** non-steroidal anti-inflammatory drugs.

* Correct answer.

### 3.3. Perceptions of community pharmacists about IBS

The survey revealed that 74.6% (n = 50) of pharmacists correctly understood IBS to be more common than colorectal cancer, with 11.9% (n = 8) disagreeing and 13.4% (n = 9) uncertain. Interestingly, 58.2% (n = 39) incorrectly believed that the prevalence of IBS exceeded that of hypertension. Most participants (91.0%, n = 60) appropriately rejected the notion that IBS exclusively affects females. Regarding disease prognosis, 25.4% (n = 17) erroneously associated IBS with increased mortality, whereas 59.7% (n = 40) correctly disagreed that IBS symptoms inevitably progressed. Additional perception data are presented in Table 3.

**Table 3:** Perceptions of community pharmacists towards irritable bowel syndrome (n = 67).

Item	Agree N (%)	Disagree N (%)	No idea N (%)
IBS or irritable bowel syndrome is so much more common than colorectal cancer.	50 (74.6)*	8 (11.9)	9 (13.4)
Compared with hypertension, IBS is more common.	39 (58.2)	13 (19.4)*	15 (22.4)
IBS only occurs in women.	8 (9.0)	81 (91.0)*	0 (0)
IBS is generally considered a condition that is associated with increased mortality	17 (25.4)	44 (65.7)*	8 (9.0)
IBS may have impact on the development of inflammatory bowel disease.	47 (70.1)*	20 (29.9)	0 (0)
IBS is among the most important risk factors for colorectal cancer.	33 (49.3)	25 (37.3)*	9 (13.4)
IBS symptoms are persistent and might get worse with time	22 (32.8)	40 (59.7)*	5 (7.5)

IBS = irritable bowel syndrome.

* Correct answer.

### 3.4. Practice and preparedness of community pharmacists about IBS

In response to clinical scenario Q1, 59.7% (n = 40) of pharmacists appropriately identified IBS and recommended specialist referral, while 35.8% (n = 24) sought additional information. For scenario Q2, 55.2% (n = 37) correctly advised gastroenterology referral, whereas 38.8% (n = 26) inappropriately suggested acetaminophen with loperamide. Only 38.8% (n = 26) selected the correct medication (Clidinium-C three times a day before meals) for stress-related IBS in scenario Q3. In Q4, 55.2% (n = 37) of participants accurately identified omeprazole as inappropriate for IBS-related bloating. The results of the complete practice assessment are presented in Table 4.

**Table 4:** Practice of community pharmacists about irritable bowel syndrome (n = 67).

Practice responses	N (%)
Q1- A 31-year-old female presents to your pharmacy complaining of bloating diarrhea, and abdominal pain, and that persist for 4 mo. She states that the abdominal pain is somewhat relieved after defecation. Currently, she is experiencing nonbloody diarrhea. Which of the following is the most appropriate action to be taken?	
a. This patient most likely has IBS; hence, should be referred to a specialist.	40 (59.7)*
b. This female does not have IBS; hence, loperamide should be prescribed.	1 (1.5)
c. Give this patient loperamide since she has been presented with IBS.	2 (3.0)
d. Inadequate patient’s data is given to take a history and more questions must be asked.	24 (35.8)
Q2- A 30-year-old female patient presents to your pharmacy with a history of complications of IBS for 3 years. She describes her chief complaints as abdominal pain, fever, and diarrhea. Which would be the best recommendation for this patient?	
a. Recommend paracetamol with dicyclomine	0 (0.0)
b. Recommend paracetamol with loperamide	26 (38.8)
c. Recommend paracetamol with clidinium-C	4 (6.0)
d. Refer this female patient to a gastroenterology specialist	37 (55.2)*
Q3- A 32-year-old male has occasional constipation and abdominal pain, especially when under stress. Which of the following drugs, if ordered by the physician would be the best choice?	
a. Amitriptyline 10 mg once daily at bedtime	4 (6.0)
b. Citalopram 20 mg once daily at bedtime	31 (46.3)
c. Alprazolam 0.5 mg once daily at bedtime	6 (9.0)
d. Clidinium-C, 3 times a day before meals	26 (38.8)*
Q4- Which one of the following is not indicated for bloating and abdominal pain in IBS patients?	
a. Hyoscine	15 (22.4)
b. Clidinium-C	15 (22.4)
c. Omeprazole	37 (55.2)*
d. Dicyclomine	0 (0.0)
Q5- A patient comes to your community pharmacy with a prescription of Asacol, loperamide, and prednisolone. Having this information, what is the most likely diagnosis among the following?	
a. IBS	25 (37.3)
b. IBD	29 (43.3)*
c. Peptic ulcer	7 (10.4)
d. Indigestion	6 (9.0)
Q6- Which of the following is suitable for use in the management of IBS?	
a. Ciprofloxacin	8 (11.9)
b. Azithromycin	14 (20.9)
c. Clindamycin	3 (4.5)
d. Metronidazole	42 (62.7)*
Q7- IBS is selective for which of the following?	
a. Garlic	2 (3.0)
b. Clopermin	36 (53.7)*
c. Anethom	0 (0.0)
d. Senna	29 (43.3)

IBD = inflammatory bowel disease, IBS = irritable bowel syndrome.

* Correct answer.

### 3.5. Awareness and perception scores of pharmacists working in community pharmacies

Using the median categorization, 61.2% (n = 41) of pharmacists demonstrated adequate awareness, while 38.8% (n = 26) had inadequate awareness. Regarding perceptions, 64.2% (n = 43) demonstrated appropriate perceptions compared with 35.8% (n = 24) who had less appropriate perceptions.

### 3.6. Associations between pharmacists’ awareness, perceptions, and demographic characteristics

The associations of pharmacists’ awareness and perceptions with years of experience and education level were evaluated (Table 5). No statistically significant associations were found between awareness (*P* = .42) or perceptions (*P* = .32) and years of experience. However, awareness was significantly associated with educational level (*P* = .041), whereas no such association existed for perceptions (*P* = .64).

**Table 5:** Associations between pharmacists’ awareness and perceptions with years of experience and education level (N = 67).

Variables	Correct responses of awareness		Correct responses of perceptions	
	Mean rank	*P*-value	Mean rank	*P*-value
Years of experience				
1–5 (n = 46)	33.59	.42	34.93	.32
6–10 (n = 15)	31.53		28.27	
>10 (n = 6)	43.33		41.17	
Level of education				
Diploma (n = 3)	57.00	.041	42.33	.64
Bachelor’s (n = 63)	32.54		33.44	
Master’s (n = 1)	57.00		44.00	

Similarly, the associations between sex and nationality were analyzed (Table 6). Neither awareness (sex: *P* = .47; nationality: *P* = .36) nor perceptions (sex: *P* = .91; nationality: *P* = .86) varied significantly according to these demographic factors.

**Table 6:** Associations between pharmacists’ awareness and perceptions with sex and nationality (N = 67).

Variables	Correct responses of awareness		Correct responses of perceptions	
	Mean rank	*P*-value	Mean rank	*P*-value
Sex				
Male (n = 42)	32.71	.47	34.20	.91
Female (n = 25)	36.16		33.66	
Nationality				
Saudi (n = 45)	35.47	.36	33.71	.86
Non-Saudi (n = 22)	31.00		34.59	

## 4. Discussion

This study, the first to assess community pharmacist awareness, perceptions, and clinical preparedness regarding IBS management in the Qassim region, revealed that while the majority of pharmacists demonstrated adequate awareness (61.2%) and constructive perceptions (64.2%) of IBS, significant knowledge gaps persist. Notably, only 17.9% recognized important warning signs, and just over half (52.6%) showed adequate clinical preparedness. These findings highlight critical areas for targeted educational interventions to optimize the role of community pharmacists in IBS care. Most participating pharmacists (68.7%) had 1 to 6 years of practice experience, a finding consistent with Hummaida et al’s report (52.4%).^[16]^ The relevance of this investigation is underscored by the consistently reported high prevalence of IBS in Saudi Arabia, which ranges from 8% to 40% compared with the global estimate of 5% to 10%.^[3]^ This elevated burden of disease within the Kingdom makes the readiness of community pharmacists – often the first point of contact for patients – particularly critical. Several factors may explain this higher local prevalence, including methodological differences across studies, rapid dietary shifts toward Westernized foods, high rates of psychological distress, increasing sedentary lifestyles, and a strong familial predisposition reported in the region.^[3]^ These factors create a significant demand for accessible, knowledgeable frontline healthcare providers, which this study directly addresses.

Community pharmacists play a vital role in IBS management through patient education and counseling.^[17,18]^ In line with this recognized role, our results showed that 61.2% of participants demonstrated adequate awareness about IBS, while 38.8% showed inadequate understanding, aligning with the findings of Hummaida et al (58% adequate awareness).^[16]^ Importantly, 89.1% of the pharmacists correctly identified common IBS symptoms, which is crucial for their frontline role in patient care. This finding agrees with those of both Hummaida et al^[16]^ and Al-Hazmi^[4]^ and reflects a consistent strength in basic symptom recognition across multiple studies, likely due to adequate coverage of gastrointestinal symptomatology in standard pharmacy curricula.

However, only 17.9% of pharmacists recognized the warning signs of IBS, differing from the findings of Al-Hazmi (8.7%) and Hummaida et al (36.9%).^[4,13]^ This wide variation across studies (8.7–36.9%) may be attributable to differences in question phrasing, the specific warning signs assessed, or regional variations in clinical exposure and continuing education. The consistently low recognition rates across all studies, however, suggest a universal gap in training regarding “red flag” symptoms that warrant specialist referral. Such gaps in awareness may affect patient counseling about the generally benign nature of IBS and the importance of ruling out serious conditions.^[19–22]^ Despite overall adequate awareness (61.2%), pharmacists showed poor awareness of IBS medications, which was consistent with other studies,^[16,17]^ possibly due to insufficient undergraduate training on functional gastrointestinal disorders, limited pharmacy references, and the perception that IBS management is primarily dietary rather than pharmacological.

Regarding perceptions, 64.2% of pharmacists held constructive perceptions of IBS, while 35.8% demonstrated problematic perceptions. Pharmacists are accessible healthcare professionals, making their perceptions crucial for patient education.^[17,23]^ The finding that over one-third of pharmacists hold less constructive perceptions is concerning, as negative or inaccurate perceptions may influence the quality of patient interactions and the therapeutic advice provided. Notably, 58.2% incorrectly believed that IBS was more prevalent than hypertension, whereas only 19.4% correctly disagreed. Additionally, 25.4% of Qassim pharmacists mistakenly associated IBS with increased mortality, a misconception that could cause unnecessary patient anxiety and inappropriate counseling. These findings highlight the need for educational interventions to correct these misperceptions.

These perception findings differ somewhat from an Indonesian study, where about half of pharmacists showed constructive perceptions.^[24]^ This discrepancy may reflect cultural differences in health beliefs, variations in pharmacy practice scope, or differences in the perception assessment tools used across studies. Furthermore, 59.7% of our participants correctly disagreed that IBS symptoms inevitably worsen over time, in contrast to Hummaida et al’s findings (18.4% disagreement).^[16]^ This substantial difference may be explained by variations in sample characteristics, regional differences in continuing education exposure, or the increasing availability of evidence-based information regarding the favorable prognosis of IBS in recent years.

In terms of preparedness, 52.65% of pharmacists demonstrated adequate clinical preparedness for IBS management, which is slightly lower than that reported by Hummaida et al (63%).^[16]^ The suboptimal recognition of appropriate pharmacotherapy for IBS (38.8%) suggests that community pharmacists may not be fully equipped to provide evidence-based medication guidance, potentially leading to suboptimal patient outcomes and inappropriate treatment recommendations. This finding is inconsistent with the same study’s report (51%).^[16]^ The lower performance in our study may reflect differences in the specific medications assessed or variations in clinical exposure between regions. Regarding herbal treatments, 53.7% answered correctly, outperforming Hummaida et al’s findings (29.1%).^[16]^ This relative strength may reflect increasing patient interest in and pharmacist exposure to complementary and alternative medicine in community practice settings.

The consistency of our findings with previous studies – particularly regarding adequate awareness but poor recognition of warning signs and pharmacotherapy – suggests that these knowledge gaps are systemic rather than isolated. They likely stem from common factors across Saudi and regional pharmacy education programs, including limited curricular emphasis on functional gastrointestinal disorders, insufficient clinical training in differentiating IBS from organic diseases, and a lack of standardized continuing education requirements for community pharmacists. The persistent nature of these gaps across multiple studies conducted over several years indicates that current educational approaches may be inadequate and require urgent reassessment.

### 4.1. Potential implications of the study

In clinical practice, the identified gaps in pharmacists’ knowledge of IBS alarming signs (e.g., anemia) and appropriate pharmacotherapy suggest a need for enhanced training. This could improve early detection of serious conditions and ensure accurate evidence-based patient counseling and medication guidance.

For pharmacy education & policy, the results highlight an opportunity to strengthen undergraduate and continuing education curricula for community pharmacists with a focus on IBS pathophysiology, symptom recognition, and first-line management. Regulatory bodies could consider mandating such training to standardize the quality of care.

Public health & patients: By addressing these educational gaps, community pharmacists can become more effective frontline resources, potentially reducing unnecessary healthcare visits, improving patient self-management, and enhancing the overall quality of life for individuals with IBS in the region.

### 4.2. Strengths and limitations

This study has notable strengths, including being the first investigation to specifically assess community pharmacists’ preparedness for IBS management in the Qassim region of Saudi Arabia. The use of scenario-based questions to evaluate clinical practice provides a more applicable measure of preparedness beyond simple knowledge recall. However, these findings must be interpreted in the context of certain limitations. First, the exclusive focus on the Qassim region limits the generalizability of the results to all community pharmacists across Saudi Arabia, suggesting that nationwide studies are warranted. Second, reliance on a convenience sampling method may introduce selection bias, potentially rendering the sample unrepresentative of the broader pharmacist population in the region. Third, this study did not account for potentially confounding variables that may influence pharmacists’ preparedness, such as varying levels of exposure to IBS cases in daily practice, personal or family history of IBS, or prior continuing education on gastrointestinal disorders. These unmeasured factors could affect the observed associations and represent important avenues for future research. Fourth, while our analysis identified significant associations, more advanced statistical methods controlling for residual confounding (e.g., multivariate regression) could strengthen causal interpretation in future studies with larger sample sizes. Fifth, potential differences between pharmacies – such as those in high versus low patient volume areas, or pharmacies with access to specialized gastroenterology references – were not assessed and may have influenced pharmacist preparedness. Despite these limitations, this study successfully identified critical knowledge and perception gaps, offering a foundational evidence base for designing targeted educational interventions to enhance IBS care in community pharmacy settings.

## Acknowledgments

The Researchers would like to thank the Deanship of Graduate Studies and Scientific Research at Qassim University (www.qu.edu.sa) for financial support (QU-APC-2026). The researchers also extend their gratitude to all patients who participated in this study.

## Author contributions

**Conceptualization:** Mohammed Saif Anaam, Saeed Obaid Alfadly, Sulaiman Ibrahim Alsohaim, Bader Abdulah Al Harbi, Bader Theyab AL Mutairi.

**Formal analysis:** Mohammed Saif Anaam.

**Methodology:** Mohammed Saif Anaam, Khalid Siddeeg, Waleed M. Altowayan.

**Data curation:** Abdulmajeed Alqasoumi, Mohammed Almunef, Sulaiman Ibrahim Alsohaim, Waleed M. Altowayan.

**Investigation:** Abdulmajeed Alqasoumi, Suhaj Abdulsalim.

**Funding acquisition:** Mohammed Almunef.

**Validation:** Mohammed Almunef, Suhaj Abdulsalim.

**Visualization:** Suhaj Abdulsalim, Khalid Siddeeg.

**Resources:** Bader Abdulah Al Harbi, Bader Theyab AL Mutairi.

**Project administration:** Mohammed Saif Anaam, Saeed Obaid Alfadly, Abdulmajeed Alqasoumi, Khalid Siddeeg, Waleed M. Altowayan.

**Supervision:** Saeed Obaid Alfadly, Mohammed Almunef, Waleed M. Altowayan.

**Software:** Mohammed Saif Anaam, Abdulmajeed Alqasoumi, Sulaiman Ibrahim Alsohaim, Suhaj Abdulsalim.

**Writing – original draft:** Saeed Obaid Alfadly.

**Writing – review & editing:** Mohammed Saif Anaam, Waleed M. Altowayan.
